# Blocking AGE-RAGE Signaling Improved Functional Disorders of Macrophages in Diabetic Wound

**DOI:** 10.1155/2017/1428537

**Published:** 2017-10-08

**Authors:** Qi Wang, Guanya Zhu, Xiaozan Cao, Jiaoyun Dong, Fei Song, Yiwen Niu

**Affiliations:** Shanghai Burn Institute, Rui Jin Hospital, Shanghai Jiao Tong University School of Medicine, Shanghai, China

## Abstract

Advanced glycosylation end products (AGEs) accumulate in diabetic wounds. Interactions between AGEs and their receptor (RAGE) leads to dermatologic problems in diabetes. Macrophage, which plays important roles in wound healing, highly expresses RAGE. Therefore, we investigated whether RAGE-expressing macrophages might be responsible for impaired wound healing on diabetes. We used anti-RAGE antibody applied topically on diabetic wounds. After confirming that wound healing was improved in anti-RAGE antibody group compared with normal mice, our results showed that macrophages appeared insufficient in the early stage and fading away slowly in the later proliferative phase compared with the control group, which was ameliorated in anti-RAGE antibody-applied wounds. Blocking AGE-RAGE signaling also increased neutrophils phagocytized by macrophages and promoted the phenotypic switch of macrophages from proinflammatory to prohealing activities. In vitro, phagocytosis of THP-1 (M0) and lipopolysaccharide- (LPS-) induced (M1) macrophages was impaired by treatment with AGEs, while IL-4- and IL-13-induced (M2) macrophages was not. Finally, AGEs increased the proinflammatory response of M1 macrophages, while inhibiting the polarization and anti-inflammatory functions of M2 macrophages. In conclusion, inhibition of AGE-RAGE signaling improved functional disorders of macrophages in the early inflammatory phase, which promoted the healing of wounds in diabetic mice.

## 1. Introduction

Morbidity resulting from diabetes mellitus is rapidly increasing worldwide and constitutes a burden to our global society [1]. Impaired wound healing is a serious complication of this disease, and it results in severe pain and reduced quality of life. There is compelling evidence that AGEs accumulate in these wounds because of certain biochemical features associated with diabetes. They are thought to contribute significantly to the pathology associated with impaired wound healing [2–4].

RAGE is a receptor for AGEs, and it is expressed in a variety of cells. It is particularly enriched in macrophages. Recent clinical and experimental research has shown that blocking the AGE-RAGE signaling interaction enhances angiogenesis, increases granulation of tissues, and promotes faster re-epithelialization in wounds. This helps to promote diabetic wound healing [5].

Macrophages play a vital role in wound healing [6–8]. Although their exact roles remain incompletely understood, [9–12] macrophage-based therapies are beneficial for some patients, including the elderly and those with hard-to-heal wounds [13–18]. This offers a cellular target for improving wound-healing therapies. However, before macrophage-based therapies can be developed, a more complete understanding of macrophage dysfunction during wound healing is required.

AGEs accumulate in the diabetic derma and contribute to impaired wound healing, together with macrophages, which express high levels of the receptor RAGE, suggests that AGE-RAGE signaling might underlie the macrophage dysfunction that is a hallmark of impaired wound healing in diabetics. Here, we studied the functional changes of macrophages during wound healing in a diabetic mouse model. We also explored the influence of AGEs on THP-1 macrophages and their relationship with AGE-RAGE signaling. These results improve our understanding of the association between AGE-RAGE signaling and the functional dysregulation of macrophages in impaired wound healing. These findings may aid the development of macrophage-based therapies for this diabetic complication in the future.

## 2. Materials and Methods

### 2.1. Induction of Diabetes in Mice and Wounding

Male C57BL/6 mice (8–10 weeks old, 20–25 g) were obtained from the Experimental Animal Center of Rui Jin Hospital in Shanghai, China. All experimental procedures were in compliance with laboratory institutional guidelines and the National Institutes of Health Guide for the Care and Use of Laboratory Animals.

To induce diabetes, a daily intraperitoneal injection of STZ (Sigma-Aldrich, St. Louis, MO, USA) at a dose of 65 mg/kg body weight was administered for 5 consecutive days. Blood glucose measurements were performed for 8 weeks after the injections. When polyuria, polydipsia, polyphagia, weight loss, and elevated blood glucose (16.7 mmol/L) were observed, mice were deemed to be in a diabetic state.

To introduce wounds in these animals, control and diabetic mice (*n* = 72 each) were anesthetized with an intraperitoneal injection of sodium pentobarbital (60 mg/kg body weight). The animals were shaved dorsally and a depilatory agent was used to remove the remaining hair. The surgical area was washed with benzalkonium bromide. One full-thickness excisional wound (9 mm diameter) was created by a sterilized punch. Throughout the experiment, all mice were individually caged and a semipermeable transparent dressing (Tegaderm; 3M Health Care, St. Paul, MN) covered the wound, which was replaced every 2 days until day 11. Diabetic and control mice were randomly assigned to three groups in which different topical treatments were applied to the wounds (the saline group (C), the rabbit IgG isotype (Bioss, Beijing, China) group (I), and the anti-RAGE antibody (Abcam, UK) group (R)). From days 0–10 after wounding, diabetic mice were treated with saline, mouse IgG isotype, or anti-RAGE antibody every 2 days. Normal control mice were topically treated with saline at the same time intervals. Mouse IgG isotype or anti-RAGE antibody (20 *μ*g/dose each) was applied directly underneath the Tegaderm dressing. In the other group, the same volume of saline was applied as a control. A preliminary analysis suggested that topically use of anti-RAGE, rabbit IgG had no difference on wound closure rates on normal mice.

### 2.2. Analysis of Wound Healing

On days 0, 1, 3, 7, and 14 after wounding, animals were anesthetized with an intraperitoneal injection of sodium pentobarbital. The edge of the wound was traced onto a transparent plastic membrane, which was then scanned for analysis using ImageJ 1.49v software.

### 2.3. Preparation of Wound Tissue

The wound and surrounding tissues (a margin of approximately 5 mm into the unwounded skin) from animals in each group were excised on 1, 3, 7, and 14 days after wounding. The excised tissues included the subcutaneous fat underneath the wound. Samples were fixed in 10% neutral-buffered formalin and stored at −80°C for future analysis.

### 2.4. Hematoxylin and Eosin (H&E) Staining and Immunohistochemistry

Wound tissue sections (4 *μ*m thick) were then deparaffinized in xylene, rehydrated through a graded alcohol series into phosphate-buffered saline (PBS). To quantify neutrophils, sections were stained with H&E and mounted in resin. Six tissue sections from each group were randomly chosen and imaged with five fields per slice at 400x magnification using a Zeiss microscope (Axioskop 2 Plus, Germany).

For immunohistochemical analyses, sections were deparaffinized and pancreatin (1 : 3; Maxim, Fuzhou, China) for antigen retrieval was performed. Sections were incubated with rabbit polyclonal anti-AGE (1 : 10000; Abcam, UK) or mouse monoclonal anti-CD68 (1 : 400; Abcam, UK) at 4°C overnight. Following incubation with an HRP-labeled secondary antibody (Dako, Denmark), chromogenic development was performed using diaminobenzidine, and sections were then counterstained with hematoxylin. Stained cells at the wound edge were manually counted on Zeiss at 200x magnification. The images were captured using a Zeiss and processed by SPOT imaging software (Diagnostic Instruments, Sterling Heights, MI).

### 2.5. Transmission Electron Microscopy (TEM)

Tissues obtained on days 1 and 3 after wounding were fixed, dehydrated, and embedded in Araldite CY212. Ultrathin sections were stained with uranyl acetate and lead citrate and visualized by TEM (CM-120 BioTwio, Philips).

### 2.6. Immunofluorescence

Deparaffinized sections were blocked with PBS containing 10% fetal bovine serum (FBS) and then incubated with rabbit polyclonal to RAGE (1 : 1000; Abcam, UK), Alexa647-conjugated anti-CD68 (1 : 50; Santa Cruz, CA, USA), Alexa488-conjugated anti-NOS2 (1 : 50; Santa Cruz, CA, USA), or anti-CD206 (1 : 1000; Abcam, UK) antibody overnight at 4°C. After incubation with a PE-conjugated donkey anti-rabbit secondary antibody (1 : 250; Abcam, UK) at room temperature for 1 h, gold antifade reagent with DAPI (Invitrogen, Basel, Switzerland) was added. Sections were visualized using a two-photon laser scanning confocal microscope (LSM 510, Zeiss, Thornwood, NY).

### 2.7. Cell Culture

A human acute monocytic leukemia cell line (THP-1) was obtained from the Cell Bank of the Chinese Academy of Sciences. Cells were cultured at 37°C in a humidified incubator under 5% CO_2_ in RPMI 1640 medium (Hyclone, USA) supplemented with 10% FBS. To induce differentiation of macrophages, THP-1 cells were cultured in the presence of 100 ng/mL phorbol-12-myristate-13 acetate (PMA; Sigma-Aldrich, St. Louis, MO, USA) for 24 h.

### 2.8. Transfection and Simulation

Stealth RNA interference duplexes against *RAGE* were designed and synthesized (GenePharma Technologies, Shanghai, China). After differentiation, *RAGE* siRNA (50 nmol/L) was delivered into THP-1 macrophages using Lipofectamine® 3000 (Invitrogen, Basel, Switzerland) according to the manufacturer's instructions. To assess the efficiency of the transfection method, fluorescein isothiocyanate-labeled nonspecific siRNAs were used to show that up to 85% of the THP-1 macrophages were successfully transformed.

Untransfected THP-1 macrophages and THP-1 macrophages transfected with *RAGE* siRNA were cultured in RPMI 1640 with 10% FBS, RPMI 1640 with 10% FBS and bovine serum albumin (BSA, 200 *μ*g/mL), or RPMI 1640 with 10% FBS and AGEs (Abcam, UK, 200 *μ*g/mL) for 24 h. LPS (100 ng/mL; Sigma-Aldrich, US) or IL-4 and IL-13 (20 ng/mL each, R&D, US) were then added for another 24 h incubation.

### 2.9. Phagocytosis Assay

Phagocytosis was examined by assessing the cellular uptake of 2.0 *μ*m-sized FITC-latex beads (Sigma, US). Beads were incubated with cells at 37°C for 30 min. Cells were then washed with precooled PBS and analyzed via flow cytometry (FC, Beckman Coulter, US).

### 2.10. Macrophage Phenotype Analysis

Cells were removed from culture dishes using 0.25% trypsin, washed with precooled PBS, and incubated with Alexa647-conjugated mouse monoclonal anti-CD68 (1 *μ*g per 1 × 10^6^ cells; Santa Cruz, CA, USA), Alexa488-conjugated mouse monoclonal anti-NOS2 (1 *μ*g per 1 × 10^6^ cells; Santa Cruz, CA, USA), or PE-conjugated mouse monoclonal anti-CD206 (10 *μ*L/106 cells; R&D, US) for 90 min at 37°C while protected from light. Cells were subsequently washed with ice-cold PBS and analyzed by FC.

### 2.11. Western Blotting (WB)

To prepare the lysates, cells were washed with ice-cold PBS and lysed for 30 minutes in prechilled radioimmunoprecipitation assay buffer and Protease Inhibitor Cocktail (100 : 1). Then, lysed liquid was transferred to 1.5 mL tubes. After centrifugation (12,000 rpm, 20 minutes), the supernatants were collected as total cellular protein extracts, and 2.5 *μ*L was used to measure protein concentration via the Bradford method (Pierce BCA Protein Assay Kit; Thermo, US) according to the manufacturer's protocol. Protein extracts from cells were separated by 8–12% polyacrylamide gel electrophoresis and transferred onto polyvinylidene fluoride (PVDF) membranes (Millipore, MA, US). Subsequently, the PVDF membranes were probed with primary antibodies against NOS2 (1 : 1000, CST, US), CD206 (1 : 1000, CST, US), TNF-*α* (1 : 1000, CST, US), and PDGF (1 : 1000, Santa Cruz, CA, USA) at 4°C overnight. PVDF membranes were then incubated with an HRP-linked secondary antibody (1 : 10000; Kangchen, Shanghai, China) for 1 h at room temperature. Signal intensities were normalized against *β*-actin as an internal control. Finally, immune complexes were detected using the Western Blotting Luminol Reagent (Millipore, MA, USA).

### 2.12. Enzyme-Linked Immunosorbent Assay (ELISA)

TNF-*α*, IFN-*γ*, PDGF, and VEGF protein levels were measured in the protein extracts using an ELISA kit (Senxiong Biotech, Shanghai, China) according to the manufacturer's instructions.

## 3. Statistical analysis

All data were represented as the means ± SD and analyzed with SPSS for Mac 21.0 (SPSS, Chicago, IL, USA). Analysis of variance (ANOVA) and Student's *t*-test were applied to determine the statistical significance of differences between groups. Statistical significance was defined as *p* < 0.05.

## 4. Results

### 4.1. Confirmation of the Diabetic State and Expression Localization of AGEs and RAGE

All mice receiving multiple injections of STZ displayed features specific to diabetes (polyuria, polydipsia, polyphagia, weight loss, and elevated blood glucose). Body weights in the STZ-injected group dropped from 21.9 ± 1.17 g to 18.1 ± 1.32 g (*p* < 0.05). During the 8 weeks following these injections, blood glucose levels also dramatically increased from 5.4 ± 0.7 mmol/L to 27.7 ± 4.1 mmol/L (*p* < 0.05).

**Figure 1:**
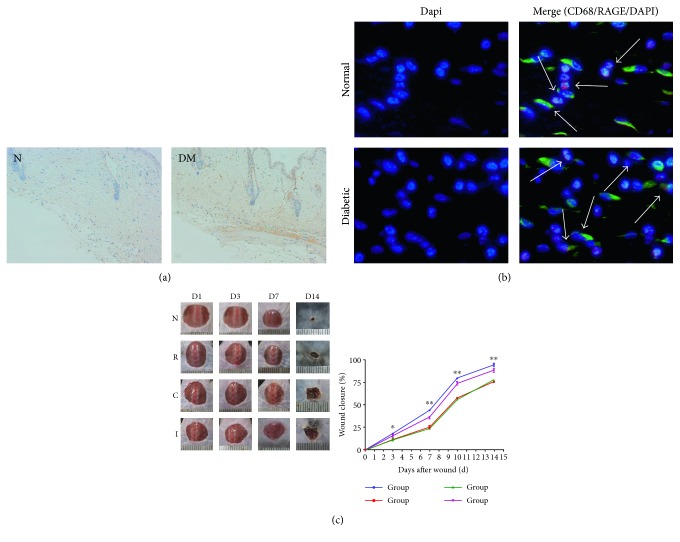
(a) Immunohistochemical staining for AGEs in the dermis of normal versus diabetic mice (200x magnification). (b) Immunofluorescence staining for colocalization of RAGE in normal versus diabetic mice (400x magnification). (c) Comparisons of the rates of wound healing among all experimental groups (*n* = 6; ^∗^*p* < 0.05, ^∗∗^*p* < 0.01).

Immunohistochemical staining for AGEs revealed faint signal in the dermal matrices and cells of control mice. In contrast, prominent signal was detected in the matrices, cells, and basement membranes of vessels of the skin in STZ-injected diabetic mice (Figure 1(a)).

To characterize the expression of RAGE on macrophages, we examined the normal and diabetic wound sections for the expression of RAGE/CD68 by immunofluorescence staining. Colocalization showed that macrophages from both normal and diabetic wound tissues expressed RAGE (Figure 1(b)).

### 4.2. Topical Application of Anti-RAGE Antibody Accelerated Diabetic Wound Healing

To examine the effects of inhibiting AGE-RAGE signaling, we confirmed that topical application of an anti-RAGE antibody accelerated wound closure in diabetic wounds. A significant difference was observed at 7 d after wounding (Figure 1(c)).

### 4.3. Blocking AGE-RAGE Interaction Improved the Removal of Neutrophils on Diabetic Mice

**Figure 2:**
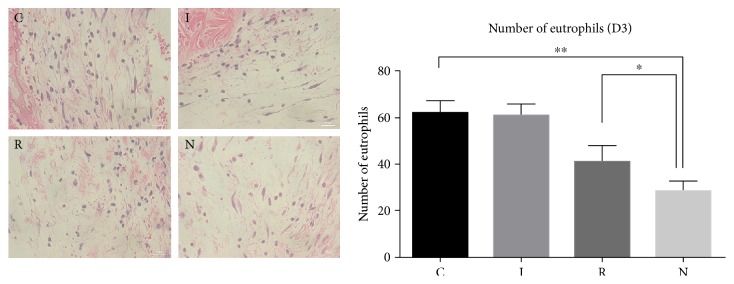
The number of neutrophils on day 3 after wounding for each experimental group (400x magnification; *n* = 6; ^∗^*p* < 0.05, ^∗∗^*p* < 0.01).

Neutrophils are the first blood-borne nucleated cells to infiltrate into injury or infection sites, where they produce multiple effector molecules [19]. As the inflammatory phase of diabetic wound healing exists, a persistence of overloaded reactive oxygen species leads to continuous damages. We examined the potential changes in neutrophil infiltration after inhibition of RAGE. We quantified the number of neutrophils present at 3 d postwounding, which is a key time point for neutrophil resolution. The amount of neutrophils significantly increased in the wounds of diabetic mice, and this was partially rescued by the application of the anti-RAGE antibody (Figure 2()).

### 4.4. AGE-RAGE Signaling Delayed Macrophage Infiltration and Neutrophil Being Phagocytized In Vivo

**Figure 3:**
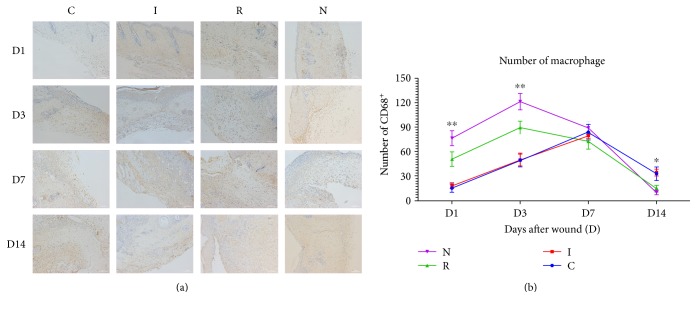
(a) Representative immunohistochemical sections showing the number of CD68^+^ cells in all experimental groups on days 1 and 14 of wound healing (200x magnification). (b) The number of CD68^+^ cells increased and then decreased slowly in the diabetic group. This was partly rescued by inhibition of AGE-RAGE signaling (*n* = 6, ^∗^*p* < 0.05, ^∗∗^*p* < 0.01).

Macrophages phagocytize apoptotic neutrophils to prevent further tissue damage, thus resulting in resolution of wound inflammation and promoting wound healing [20, 21]. After finding out that the infiltration of neutrophils was incresed in diabetic mice, which was modulated by AGE-RAGE signaling, we examined whether macrophage infiltration and phagocytosis were disrupted in diabetic mice. Macrophage infiltration was assessed by immunohistochemical staining for CD68 (Figure 3(a)). One day postwounding, group N displayed the presence of many CD68^+^ cells, while groups C and I had significantly fewer. Meanwhile, group R had a large quantity of CD68^+^ cells, but fewer than group N. At 14 d after wounding, groups N and R displayed significantly fewer CD68^+^ cells compared to groups C and I. Immunohistochemical staining for CD68^+^ macrophages suggested that inhibition of the AGE-RAGE interaction partly reversed a phenomenon described previously as “macrophages arriving slowly and fading away slowly” [8] (Figure 3(b)).

**Figure 4:**
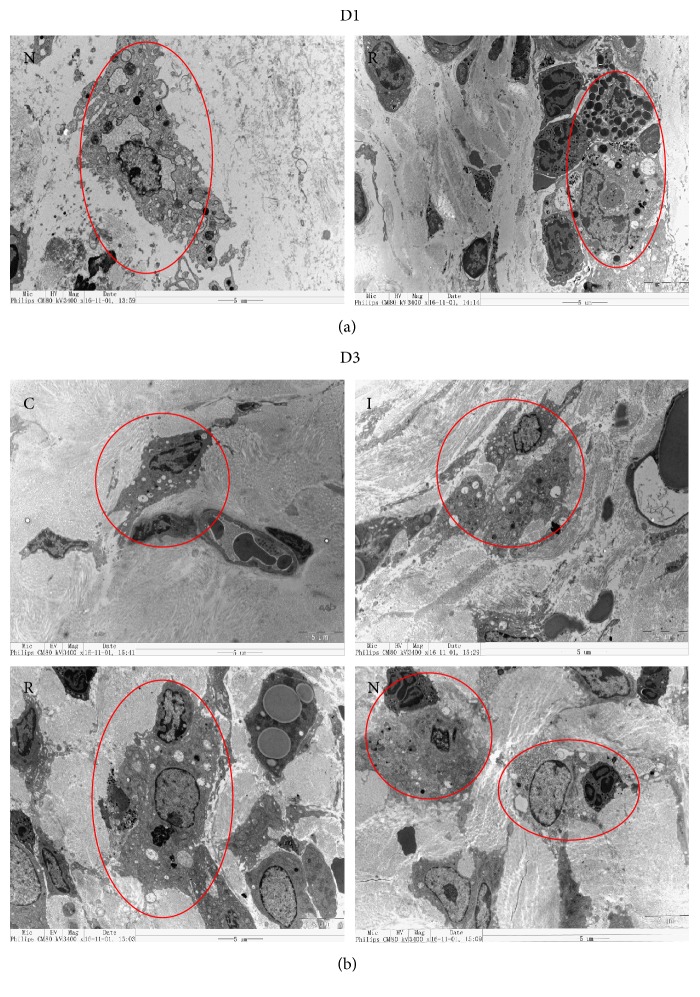
(a) TEM micrographs of wound tissue from groups R and N on day 1 of wound healing (3400x magnification). (b) Uptake of neutrophils by macrophages in groups N and R on day 3 of wound healing (3400x magnification).

Samples collected on days 1 and 3 after wounding were examined for the presence of macrophages by TEM. At 1 d, groups N and R displayed macrophage infiltration at the wound edge, while groups C and I did not (Figure 4(a)). At 3 d, groups N and R showed the expected uptake of neutrophils by macrophages at the wound edge. In contrast, groups C and I possessed macrophages that lack any signs of phagocytosis (Figure 4(b)).

### 4.5. AGE-RAGE Signaling Contributes to Polarization Disorder in Macrophages In Vivo

**Figure 5:**
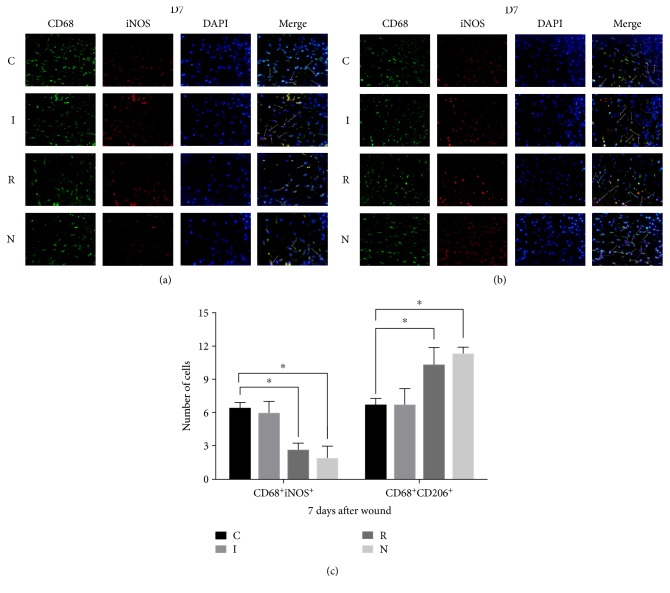
(a, b) Fluorescent staining of cells expressing CD68, iNOS, or CD206 on day 7 of wound healing compared to group N. The number of iNOS^+^ cells increased, which was rescued by anti-RAGE antibody treatment. (c) The number of cells expressing CD68, iNOS, or CD206 for each experimental group (^∗^*p* < 0.05).

Because macrophages involved in diabetic wound healing display a switch disorder between proinflammatory and prohealing phenotypes [10, 22], we examined whether blocking the AGE-RAGE interaction could reverse these defects. We tested the expression of various markers for macrophages (CD68), the proinflammatory phenotype (iNOS), and the prohealing phenotype (CD206) using immunofluorescence at 7 d postwounding, which is a critical time point during the proliferative phase (Figures 5(a) and 5(b)). Compared to group N, we observed that the number of iNOS^+^ cells increased significantly, and this was rescued by treatment with the anti-RAGE antibody (Figure 5(c)). Therefore, inhibiting AGE-RAGE signaling might reduce the switch disorder of macrophage polarization.

### 4.6. AGEs Inhibited Phagocytosis in M0 and M1 Macrophages via AGE-RAGE In Vitro

**Figure 6:**
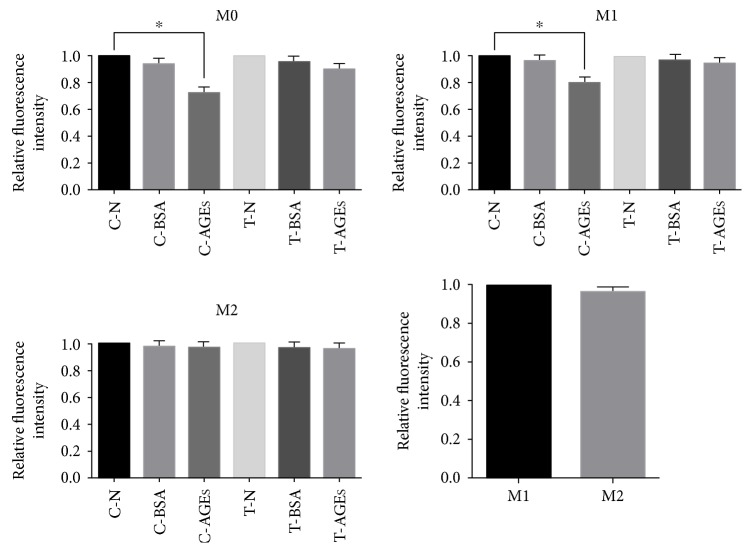
Relative fluorescence intensity of M0, M1, and M2 macrophages (^∗^*p* < 0.05). Relative fluorescence intensity of M0 and M1 macrophages decreased in the AGE treatment groups. This decrease was less significant after application of the anti-RAGE antibody.

To examine whether AGE-RAGE signaling inhibits macrophage phagocytosis, we compared the fluorescence intensities among THP-1 M0 macrophages, LPS-induced M1 macrophages, and IL-4- and IL-13-induced M2 macrophages. Surprisingly, AGEs impaired phagocytosis in M0 and M1 macrophages, but not M2 macrophages. Furthermore, phagocytosis was rescued after blocking AGE-RAGE signaling. A comparison between M1 and M2 macrophages showed that M2 macrophages displayed phagocytic rescue if provided with beads in a manner comparable to M1 macrophages (Figure 6).

### 4.7. AGEs Improved Proinflammatory Functions of M1 Macrophages While Inhibiting Polarization and Anti-Inflammatory Functions of M2 Macrophages

**Figure 7:**
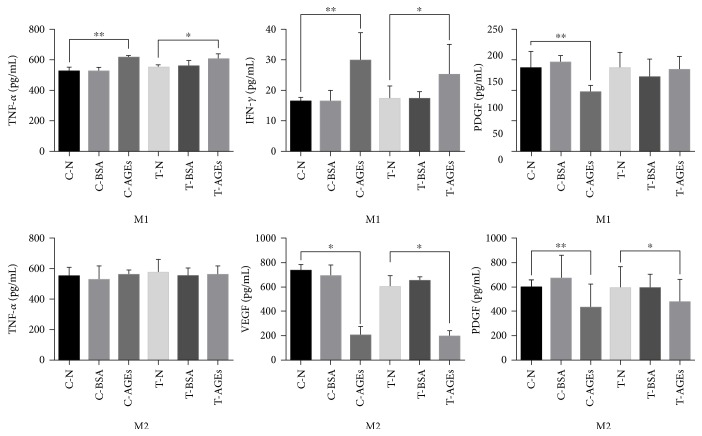
ELISA showing the secretion levels for TNF-*α*, IFN-*γ*, PDGF, and VEGF from M1 and M2 macrophages in each experimental group (*n* = 6, ^∗^*p* < 0.05, ^∗∗^*p* < 0.01).

**Figure 8:**
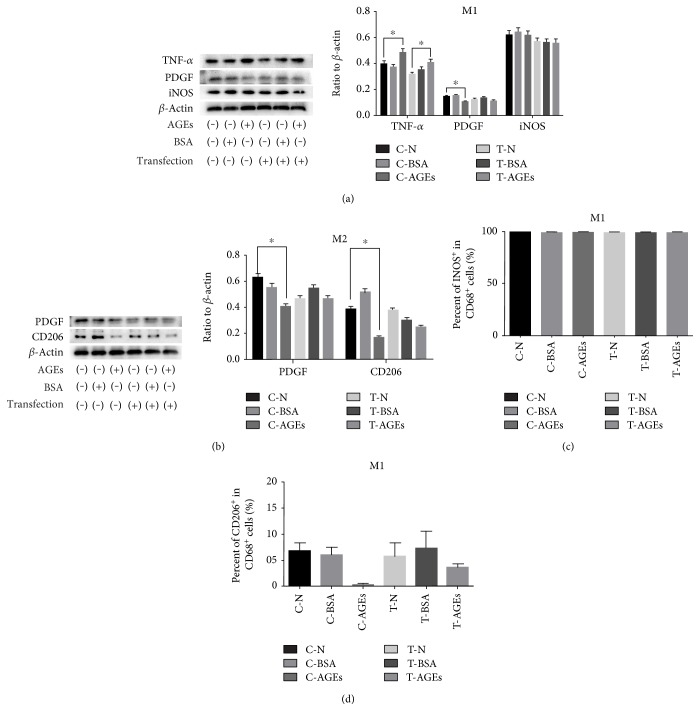
(a, b) WB showing the expression of TNF-*α*, PDGF, iNOS, and CD206 in M1 and M2 macrophages for each experimental group (*n* = 6, ^∗^*p* < 0.05). (c, d) FC analysis showing expression of iNOS and CD206 in M1 and M2 macrophages for each experimental group (*n* = 6, ^∗^*p* < 0.05).

To test the influence of AGE-RAGE on macrophage secretion and polarization, we performed ELISA and WB. The outcome of ELISA showed that M1 macrophages had higher TNF-*α* and IFN-*γ* secretion levels and a lower PDGF secretion level, which correlated with AGE-RAGE. For M2 macrophages, AGEs impaired the secretion of VEGF and PDGF by RAGE, while no difference was observed for TNF-*α* secretion (Figure 7). WB showed that AGE-RAGE signaling improved the TNF-*α* secretion of M1 macrophages, and these M1 macrophages also displayed the ability to secrete the growth factor PDGF. PDGF is typically decreased in the presence of AGEs, but to a lesser extent after blocking AGE-RAGE. However, the expression of the M1 macrophage marker iNOS was not affected among these groups (Figure 8(a)). With respect to M2 macrophages, AGE-RAGE signaling inhibited the expression of the M2 marker CD206 and PDGF secretion (Figure 8(b)).

By FC analysis, we detected the expression of CD68, iNOS, and CD206 in M1 and M2 macrophages. More than 99% of the CD68^+^ cells expressed iNOS in all six experimental groups (Figure 8(c)). In addition, the expression of CD206 in M2 macrophages was downregulated after stimulation with AGEs, which was rescued by blocking the AGE-RAGE interaction (Figure 8(d)).

## 5. Discussion

Based on the results in vivo and in vitro, we found that AGE-RAGE signaling correlated with the disruption of macrophage function, including inhibition of phagocytosis and cytokine secretion. These processes play vital roles in the resolution of local inflammation and wound healing. Perturbing these functions may disrupt the healing process in diabetic wounds of human patients. Furthermore, we suggest that the early phase of the inflammatory process is an ideal control point for targeting macrophage-based therapies.

A disease-associated microenvironment state marked by AGEs accumulation in diabetes has been reported by multiple lines of evidences [2], which indicates that the AGE-RAGE interaction contributes to failed wound healing on diabetes. First of all, the accumulation of AGEs and the ability of the anti-RAGE antibody to block the AGE-RAGE interaction were confirmed in our STZ-induced diabetic mouse model. Oxidative stress is thought to occur during the early inflammatory phase of diabetic wounds, and these lesions are often described as being “stuck” in the inflammatory phase [21]. Consistent with this, we found that AGE-RAGE signaling devoted to insufficient macrophages infiltration and phagocytized neutrophils on diabetic mice. In addition to a deficiency in the numbers of macrophages, we also explored whether phagocytosis in macrophages normally devoted to clearing neutrophils was affected by AGEs. Using an in vitro phagocytosis assay, we found that AGEs impaired phagocytosis in THP-1 macrophages and LPS-induced M1 macrophages via AGE-RAGE signaling. Interestingly, IL-4- and IL-13-induced M2 macrophages displayed similar levels of phagocytosis as those observed in M1 macrophages, which were not affected by AGEs. Ignoring the polarization phenotypes that we observed, these cells were clearly all of the macrophage identity, regardless of whether they were induced by particular cytokines. This explains why M2 macrophages also display similar phagocytic dynamics compared to M1 macrophages. We suggest that when M2 macrophages participate in wound healing, the secretion of various factors aid proliferation and regeneration. As for these macrophages' resistance to the effects of AGEs, this warrants further research. In vivo and in vitro experiments clearly showed that AGE-RAGE signaling impaired macrophage phagocytosis, which is important for shutting down inflammatory responses during wound healing.

Macrophages are known to switch from a proinflammatory phenotype to a wound-healing phenotype to resolve inflammation and initiate the healing process. However, macrophages in diabetic wounds undergo a switch disorder between inflammation and healing [23–25]. By immunofluorescence, we found that blocking the AGE-RAGE interaction improved defects in macrophage polarization, which were persistent in M1 macrophages. This also served to delay the appearance of M2 macrophages in vivo. To further establish that disruptions in macrophage function are related to AGE-RAGE signaling, polarization and cytokine secretion levels were also assessed in vitro. WB and ELISA results suggested that AGE-RAGE signaling improves the proinflammatory functions of M1 macrophages and impairs the anti-inflammatory functions of M2 macrophages. Meanwhile, the expression of M2 macrophage markers suggests that AGE-RAGE interactions inhibit the polarization of these cells. Furthermore, the expression of an M1 macrophage marker did not differ among the six experimental groups by WB. Meanwhile, FC analysis found that over 99% of the THP-1 macrophages expressed M1 macrophage markers after stimulation by LPS. That may explain why WB analysis detected no differences in expression levels among the test groups. Additionally, since there were a roughly equivalent number of M1 macrophages, the increase in expression of inflammatory factors by WB indicates that AGE-RAGE signaling improves the ability of cells to secrete these factors, which might be the consequence of impaired phagocytosis.

Interestingly, WB and ELISA detected the secretion of the growth factor PDGF in M1 macrophages, while M2 macrophages secreted another inflammatory factor, TNF-*α*. Although few in numbers, FC analysis identified some macrophages that coexpressed CD68, iNOS, and CD206. Other investigators have suggested that wound-specific macrophages possess hybrid M1/M2 activation phenotypes that make them highly plastic, conferring the ability to switch between inflammatory and wound healing functions [26–28]. All these observations indicate that there are limitations to the M1-M2 definition. All these facts proved that M1-M2 definition by stimuli encountered in vitro had its limitation to apply in vivo. What is more is that as the proliferation phase has been hindered by functional disorders of macrophages in the early inflammatory phase, it is unrealistic to figure out the proper time points and interventions. Thus, in complex and dynamic cellular microenvironments, the early inflammatory phase during wound healing is a more convenient and ideal control point for macrophage-based therapies compared to the proliferation phase.

## 6. Conclusions

Taken together, the current study demonstrates that blocking the AGE-RAGE interaction improves the function of macrophages, thus promoting diabetic wound healing. Unfortunately, we could not identify the specific functional dysregulations of macrophages in vivo, and this remains a pressing topic for future research. Regardless, our findings suggest that there is a close relationship among AGE-RAGE, wound healing, and macrophage physiology. Additionally, this study offers insights that could help develop therapies that target early-acting, wound-specific macrophages in patients suffering from diabetic lesions. Future work should focus on the mechanisms controlling functional dysregulation of macrophages during wound healing in vivo.
